# Adaptive value of foot-and-mouth disease virus capsid substitutions with opposite effects on particle acid stability

**DOI:** 10.1038/s41598-021-02757-3

**Published:** 2021-12-06

**Authors:** Flavia Caridi, Rodrigo Cañas-Arranz, Ángela Vázquez-Calvo, Patricia de León, Katherine I. Calderón, Esteban Domingo, Francisco Sobrino, Miguel A. Martín-Acebes

**Affiliations:** 1 Centro de Biología Molecular “Severo Ochoa” (CSIC-UAM), 28049 Madrid, Spain; 2 Department of Biotechnology, Instituto Nacional de Investigación y Tecnología Agraria y Alimentaria (INIA, CSIC), 28040 Madrid, Spain

**Keywords:** Antivirals, Viral evolution

## Abstract

Foot-and-mouth disease virus (FMDV) is a picornavirus that exhibits an extremely acid sensitive capsid. This acid lability is directly related to its mechanism of uncoating triggered by acidification inside cellular endosomes. Using a collection of FMDV mutants we have systematically analyzed the relationship between acid stability and the requirement for acidic endosomes using ammonium chloride (NH_4_Cl), an inhibitor of endosome acidification. A FMDV mutant carrying two substitutions with opposite effects on acid-stability (VP3 A116V that reduces acid stability, and VP1 N17D that increases acid stability) displayed a rapid shift towards acid lability that resulted in increased resistance to NH_4_Cl as well as to concanamicyn A, a different lysosomotropic agent. This resistance could be explained by a higher ability of the mutant populations to produce NH_4_Cl-resistant variants, as supported by their tendency to accumulate mutations related to NH_4_Cl-resistance that was higher than that of the WT populations. Competition experiments also indicated that the combination of both amino acid substitutions promoted an increase of viral fitness that likely contributed to NH_4_Cl resistance. This study provides novel evidences supporting that the combination of mutations in a viral capsid can result in compensatory effects that lead to fitness gain, and facilitate space to an inhibitor of acid-dependent uncoating. Thus, although drug-resistant variants usually exhibit a reduction in viral fitness, our results indicate that compensatory mutations that restore this reduction in fitness can promote emergence of resistance mutants.

## Introduction

Whether a trade-off exists between capsid stability and evolvability is an important issue for viral evolution. Viral capsids protect the genetic material from environmental insult to warrant functional integrity of the viral genome^1^. However, capsids are not static containers as they are also subjected to multiple selective pressures that include immune responses, receptor recognition, physicochemical factors or antiviral drugs. Accordingly, capsid proteins are under continuous evolution leading, among others, to extensive antigenic variation, generation of drug-resistant mutants or adaptation to unfavorable environmental conditions. This evolutionary potential is constrained by the need for a balance between functionality and stability because capsids have to be stable enough to protect the genetic material while permitting its release within target host cells.

Picornaviruses are simple models to study the evolution of viral capsids. Its icosahedral capsid is formed by 60 copies of each of the four structural proteins (VP1–VP4) arranged into 12 pentameric subunits^2^. Interestingly, picornavirus capsids differ remarkably in their sensitivity to acidic pH, ranging from the resistance of *Enterovirus* to the extreme acid-lability of the *Aphthovirus* foot-and-mouth disease virus (FMDV)^3^. FMDV is the etiological agent of a highly devastating disease of cloven-hoofed animals that affects important livestock species such as swine, cattle, goats or sheep^4^. As described for other viruses, the acid-lability of FMDV capsid has been related to its mechanism of uncoating inside cellular endosomes^5,6^. The current model supports that acidic pH inside endosomes triggers FMDV capsid dissociation into pentameric subunits^7^. This may be produced by electrostatic repulsions originated from the protonation of key histidine residues at pHs slightly below neutrality^8^. Thus, neutralization of endosomal pH using ammonium chloride (NH_4_Cl), -which acts as proton sink within endosomes impairing their acidification-, inhibited FMDV infection by preventing virus uncoating^9–12^. Accordingly, the relation between acid-dependent uncoating and sensitivity to NH_4_Cl has provided an interesting model for the study of the effects of a host-targeting antiviral on viral evolution^11,13^.

The quasispecies organization of FMDV implies that viral populations are complex and dynamic distributions of variants with a huge potential for variation^14^. This has enabled the isolation from FMDV mutant distributions of a broad panel of mutant FMDVs differing in their sensitivity to acidic pH, shedding light on the molecular mechanisms of FMDV uncoating^12,13,15–20^. Remarkably, the capsid of FMDV displays a high degree of plasticity to tolerate mutations, and provides a good model system for the study of the genetic mechanisms that contribute to viral evolution^21–28^. Mutations affecting capsid stability have provided evidence for multifunctional residues^18^, the existence of additive effects^13,18,29^, or the accommodation of compensatory mutations to restore viral fitness^25,30,31^, supporting that the stability of FMDV capsid is a multifactorial trait due to the interaction between residues from different capsid proteins^13^.

In this work, we have analyzed the relationship between acidic pH sensitivity and resistance to inhibition of endosome acidification using NH_4_Cl. Our results show that the combination of two amino acid substitutions with opposite effects on acid-stability resulted in an increase in viral fitness. Such an increase mediated the adaptation to the inhibition of endosomal acidification exerted by NH_4_Cl through rapid selection of resistant variants.

## Materials and methods

### Viruses, infections and titrations

All the FMDV variants were recovered from their respective infectious cDNA clones derived from pMT28 plasmid, which contains the complete genomic sequence of the type C FMDV isolate C-S8c1 (wild type, WT)^32^. The viruses used in this study were the WT FMDV, the single mutants VP1 V11I, VP1 N17D, VP2 H145Y, VP3 A116V, and VP3 A118V; the double mutants VP2 H145Y + VP1 N17D, VP3 A116V + VP1 N17D, and VP1 N17D + VP1 T12A; and the triple mutants VP3 A116V + VP1N17D + VP1 T12A, and VP3 A116T + VP3 A118V + VP1 N17D. The origin of the mutations and the procedures followed for infectious cDNA clone manipulation, in vitro transcription and RNA-transfection have been previously described^11–13,18^. To minimize effects of viral amplification on the mutant spectrum composition, viruses were directly harvested at 48 h after transfection of BHK-21 cells with the corresponding in vitro synthesized viral RNA. The identities of the consensus sequence of the viral populations recovered from transfections were verified by nucleotide sequencing^11^. Unless otherwise specified, viral stocks were produced by transfection of in vitro synthesized RNA and two serial passages of the virus recovered. Procedures for infections in liquid medium and virus titration in semisolid agar medium were as described^10,11^. Virus titer was calculated as the number of plaque-forming units (PFU)/mL by standard plaque assay using semisolid agarose medium. The multiplicity of infection (MOI) defined as the number of PFU/cell in each experiment is indicated in the corresponding figure legend.

### Acid-inactivation assays

Procedures for determination of acid-sensitivity of FMDV have been previously described^11,33^. Briefly, equal number of PFU from each population were treated with PBS buffer adjusted to different pHs for 30 min, then the buffer was neutralized and the remaining infectivity in each sample was determined by standard titration in BHK-21 cells.

### Inhibitors and treatments

Inhibition of endosome acidification using NH_4_Cl (Merck, Darmstadt, Germany) was performed as described^11^. Cells were incubated in culture medium containing 25 mM NH_4_Cl (plus 25 mM HEPES at pH 7.4 to buffer extracellular pH) from 1 h prior to infection and throughout the rest of the assay. For inhibition of endosome acidification using concanamycin A, cells were treated for 30 min before infection with 100 nM concanamycin A (Sigma; St. Louis, MO) and the drug was maintained only during the first hour of infection^11^. Control cells were treated in parallel with drug vehicle (DMSO). Treatment with guanidinium hydrochloride (GuHCl; Sigma) was performed as described^34^. One hour prior to infection cells were incubated with 4 mM GuHCl that was maintained for the rest of the experiment.

### Next-generation sequencing (NGS)

Cells were infected in the presence or in the absence of NH_4_Cl (MOI of 0.01 PFU/cell) in triplicate wells. Viral RNA was extracted from each sample and cDNA was synthesized. A fragment of 463 nucleotides in length comprised between the C terminus of VP3 and the N terminus of VP1 was amplified using oligonucleotide primers CGGGACAATCAACCTACAC GTTGGTTATCCGACACTG coupled to nucleotide adapters CS1/CS2. Library preparation, amplification and sequencing were performed at the Parque Científico de Madrid using a MiSeq equipment (Illumina, San Diego, CA, USA) using the 2 × 300 bp MiSeq Reagent Kit v3. A total of 6231577 were analyzed (average reads per sample 519,298 ± 122,251).

### Biological fitness assessment by competition experiments

Equal number of PFU of FMDV WT and FMDV with substitutions A116V in VP3 and N17D in VP1 (abbreviated VP3 A116V + VP1 N17D) populations were mixed (initial MOI of 0.1 PFU/cell [0.05 for each virus]) and used to infect BHK-21 cells treated or not with NH_4_Cl. Viruses recovered from these infections were harvested and further passaged 10 times under the same conditions. The nucleotide sequence of the capsid coding region was determined by cDNA synthesis and automated nucleotide sequencing as described^12^. The proportions of the competing genomes were estimated from the chromatograms as a ratio of the integrated areas of each nucleotide under each peak of mutated position^12,30^.

### Statistics

Data analysis was performed using GraphPad PRISM 7 for Windows (GraphPad Software Inc., San Diego, CA, USA). Two tailed Student’s t test *P* values between control and drug-treated samples were corrected for multiple comparisons using the Sidak–Bonferroni method. Differences among viruses were analyzed by one-way analysis of the variance applying Bonferroni’s correction for multiple comparisons. Asterisks in the figures denote *P* values (*P < 0.05 and **P < 0.005). Unless otherwise specified, data represent the means ± standard deviations (SDs). The number of independent experiments analyzed (*n*) in each case is indicated in the corresponding figure legend.

## Results

### Double FMDV mutant VP3 A116V + VP1 N17D does not conform to the canonical relationship between uncoating pH and resistance to NH_4_Cl

The uncoating of FMDV is triggered by acidification inside endosomes where viral particles are delivered following the endocytic route. A variety of studies support a correlation between acid-lability and resistance to the endosomal acidification blockage induced by NH_4_Cl^7^. Accordingly, FMDVs with increased acid lability display higher resistance to NH_4_Cl^11,20^. In contrast, mutants with increased acid stability display increased sensitivity to NH_4_Cl^12,17,19^.

**Figure 1:**
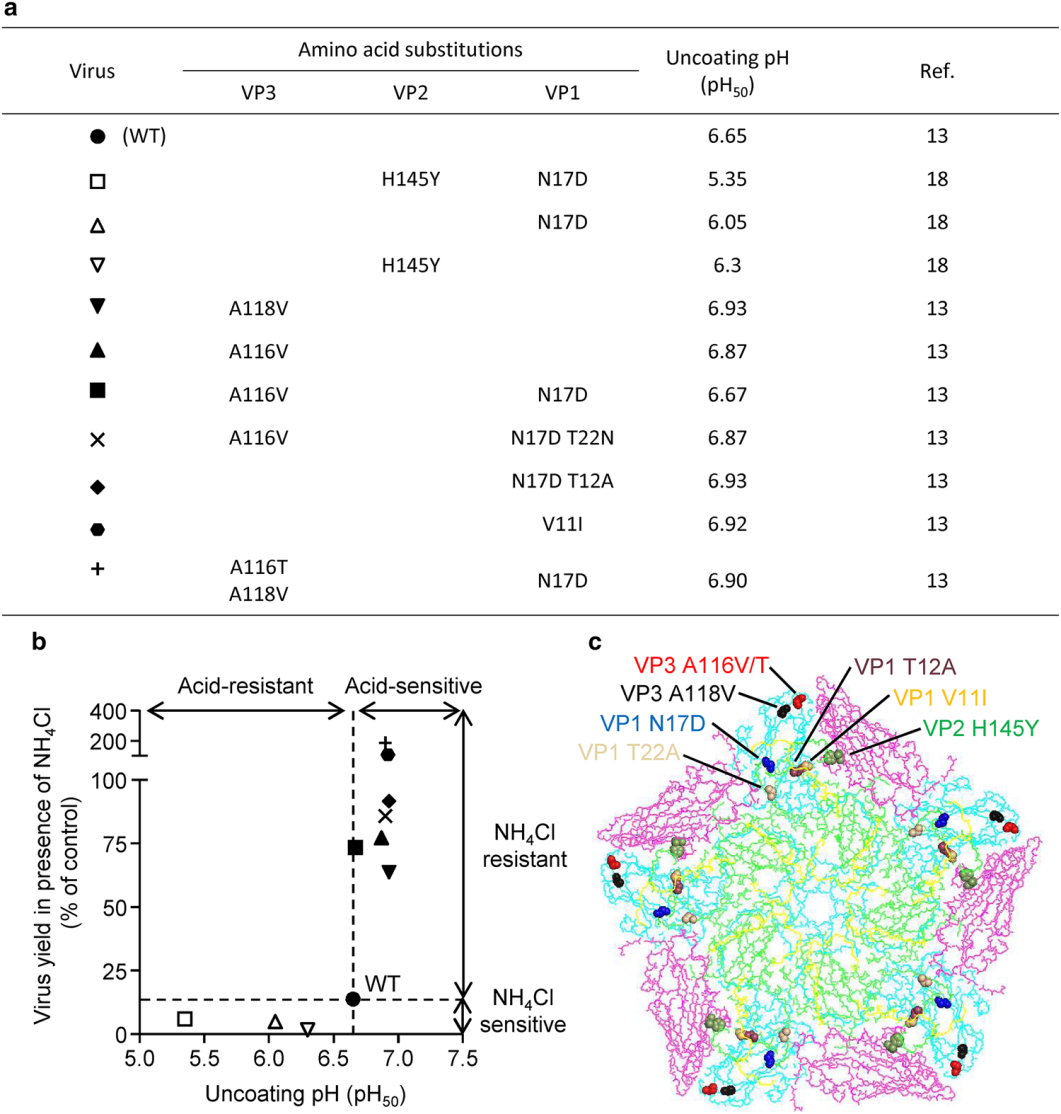
Double FMDV mutant with VP3 A116V and VP1 N17D does not follow the relation between uncoating pH and resistance to NH_4_Cl in FMDV. (**a**) Combinatorial mutants analyzed in the study. Symbols in the first column identify Each mutant, with the amino acid substitutions, the uncoating pH (pH_50_) and references included in the columns on the right. See text for details. (**b**) Relation between uncoating pH and sensitivity to endosomal neutralization by NH_4_Cl of FMDV mutants^13,18^. FMDV mutants are identified with the symbols explained in (**a**). BHK-21 cells treated or not with 25 mM NH_4_Cl were infected (MOI of 0.5 PFU/cell) with FMDV C-S8c1 (WT) or its variants. Virus yield obtained in samples treated with NH_4_Cl was determined at 8 h post-infection, and is expressed as a percentage of that obtained in samples not treated with the drug. Mean virus yield (*n* = 3) of each mutant was plotted as a function of its uncoating pH, estimated by pH_50_ value. (**c**) Location on the structure of FMDV C-S8c1 capsid^44^ of amino acid substitutions present in the mutants analyzed. An inside schematic view of a pentameric subunit is displayed. Only amino acid main chains are shown, for clarity. VP1 is green, VP2 is magenta, VP3 is cyan, and VP4 is yellow.

To systematically test this hypothesis, we analyzed the sensitivity to NH_4_Cl of 10 FMDV variants (Fig. 1A) differing in their uncoating pH (estimated by pH_50_, defined as the pH value that results in a 50% loss of infectivity) (Fig. 1B). These viruses included wild type (WT, pH_50_ 6.65), acid-resistant (pH_50_ < 6.65) and acid-sensitive viruses (pH_50_ > 6.65). The panel of viruses analyzed carried amino acid replacements in regions that have been proposed to modulate FMDV capsid stability through different mechanisms (Fig. 1C). These substitutions included: (1) bulkier residues in VP3 located close to the pentameric interface that destabilize inter pentameric interactions, destabilize viral capsid and increase its acid lability (A116V, A116T, and A118V)^11,13^; (2) amino acid replacements located in the N terminus of VP1 (N17D, T22N, T12A, and V11I)^12,13^ that regulate acid stability by a not well characterized mechanism in which only the relevance of the electrostatic interaction with negatively charged RNA has been identified for VP1 N17D mutant^35^; (3) the amino acid replacement VP2 H145Y located near the intra protomeric interface, region key for *Aphthovirus* stability^36,37^, which has been also shown to increase acid resistance in several FMDV serotypes^17,18^. As expected, the yield of acid-resistant viruses was more inhibited by NH_4_Cl than that of the WT. Acid-sensitive viruses were less susceptible or even fully resistant to the drug (Fig. 1B). Surprisingly, although its acid sensitivity (pH_50_, 6.67) was similar to that of the WT, FMDV VP3 A116V + VP1 N17D showed a behavior very similar to that of acid-sensitive viruses, with increased resistance to NH_4_Cl in comparison to WT. Thus, this mutant deviates from the correlation between acid sensitivity and resistance to the inhibition of acid-dependent uncoating by means of endosomal acidification blockage with NH_4_Cl.

### Increased resistance of double FMDV mutant VP3 A116V + VP1 N17D against another inhibitor of endosomal acidification

**Figure 2:**
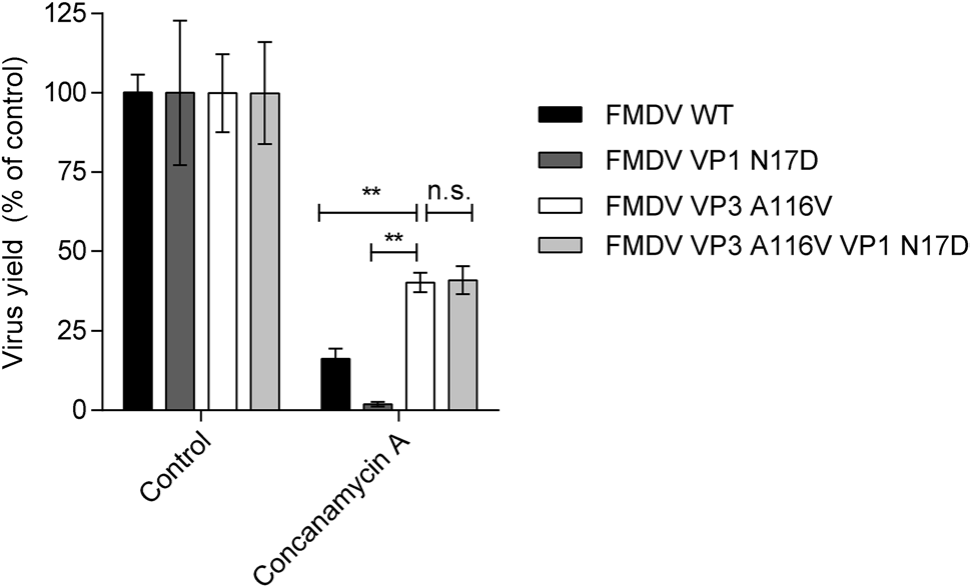
Double FMDV mutant VP3 A116V + VP1 N17D also displays increased resistance to the inhibitor of endosome acidification Concanamycin A. Analysis of the resistance of FMDV WT and FMDV VP3 A116V + VP1 N17D to Concanamycin A. BHK-21 cells treated or not with 100 nM Concanamycin A, were infected with FMDV FMDV VP3 A116V VP1 N17D, single mutants VP3 A116V, VP1 N17D or WT at a MOI of 0.5 PFU/cell. Virus yield at 8 h post-infection was determined by standard plaque assay. Two tailed Student’s t-test *P* values between double mutant and the rest of viruses were corrected for multiple comparisons using Bonferroni’s method (**P < 0.005; n.s. non-significant). Data represent the means ± SDs (n = 2–3).

The effect of Concanamycin A, an inhibitor of vacuolar-ATPase that blocks endosome acidification, was also analyzed on the infection of FMDV mutant VP3 A116V + VP1 N17D (Fig. 2). A significant increase in the resistance of FMDV mutant VP3 A116V + VP1 N17D against Concanamycin A was noticed when compared to WT and single mutant VP1 N17D, thus displaying a behavior similar to that of single mutant VP3 A116V. These results confirmed that the resistance of FMDV mutant VP3 A116V + VP1 N17D against endosome acidification applies also to inhibitors other than NH_4_Cl.

### Treatment with NH_4_Cl promotes a quick acid-lability shift of FMDV VP3 A116V + VP1 N17D

**Figure 3:**
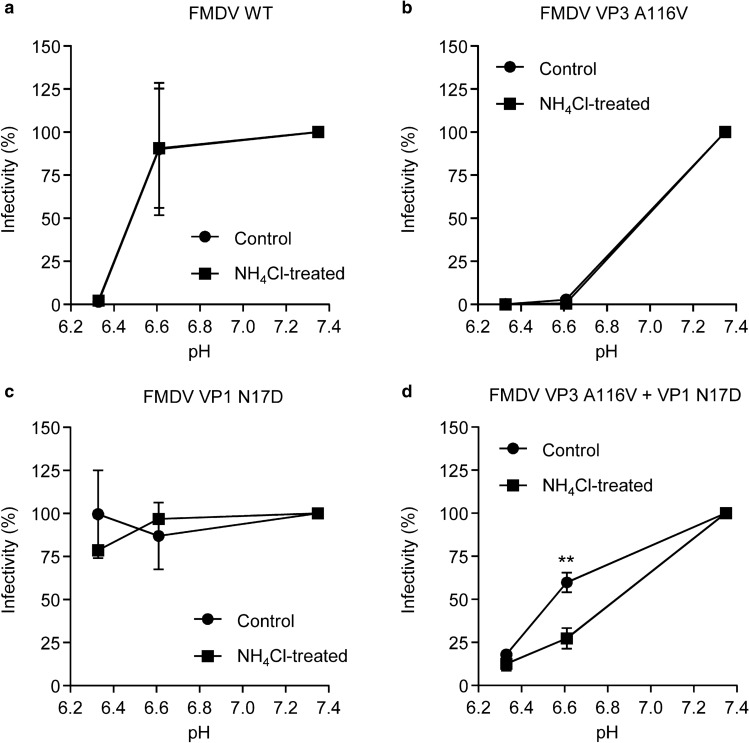
Acid-lability shift of FMDV VP3 A116V + VP1 N17D population after a single passage in the presence of NH_4_Cl. Acid sensitivity profiles of WT (**a**), VP3 A116V (**b**), VP1 N17D (**c**), and double mutant VP3 A116V + VP1 N17D (**d**) after a single amplification in the presence of NH_4_Cl. Viruses were grown in the presence or absence of 25 mM NH_4_Cl (MOI of 0.5 PFU/cell) for 8 h, and the acid sensitivity of the progeny populations was analyzed. For this purpose, equal number of PFU from each population were treated with PBS at pH 7.3, 6.6 and 6.3 for 30 min (abscissae). The pH was neutralized, and the remaining PFU in each sample was determined in BHK-21 cells. Infectivity was calculated as the percentage of PFU recovered at each different pH relative to that obtained at pH 7.3 (ordinate). Two tailed Student’s *t*-test *P* values between control and NH_4_Cl-treated populations were corrected for multiple comparisons using the Sidak–Bonferroni method (**P < 0.005). Data represent the means ± SDs (*n* = 3).

The virus carrying amino acid replacements A116V in VP3 and N17D in VP1 is a rationally designed double mutant that combined a determinant of acid sensitivity (VP3 A116V) and a determinant of acid resistance (VP1 N17D) in the same genome, yielding a virus with similar acid sensitivity than FMDV WT^13^. Searching for an explanation of the unexpected resistance of FMDV VP3 A116V + VP1 N17D to NH_4_Cl (Fig. 1b), we compared the inactivation profiles at different acidic pHs of this mutant, the corresponding single mutants, and FMDV WT, grown in the presence or in the absence of NH_4_Cl. To this end, we analyzed the acid sensitivity of the infection progenies recovered at 8 h post-infection. Viruses were incubated in buffers adjusted to different pH values and the remaining infectious virus was determined by plaque assay (Fig. 3a–d). No significant changes in the acid inactivation profiles were noted in the presence of NH_4_Cl for FMDV WT or the single mutants (Fig. 3a–c). However, the NH_4_Cl-treatment was sufficient to produce a significant decrease in the infectivity recovered upon incubation of FMDV VP3 A116V + VP1 N17D at a pH of 6.6, the uncoating pH of FMDV WT, when compared with progeny production in absence of NH_4_Cl (Fig. 3d). Thus, these results indicate that the augmented resistance to NH_4_Cl of FMDV VP3 A116V + VP1 N17D is concomitant with a rapid increase in acid lability of the viral population in response to NH_4_Cl treatment.

To analyze the genetic determinants of the altered NH_4_Cl sensitivity, the consensus sequence of the capsid-coding region of the NH_4_Cl-treated population of FMDV VP3 A116V + VP1 N17D was determined. No changes in this sequence were observed suggesting that no additional amino acid replacements were responsible for this behavior.

### Increased frequency of NH_4_Cl–resistant variants in FMDV VP3 A116V + VP1 N17D populations

**Figure 4:**
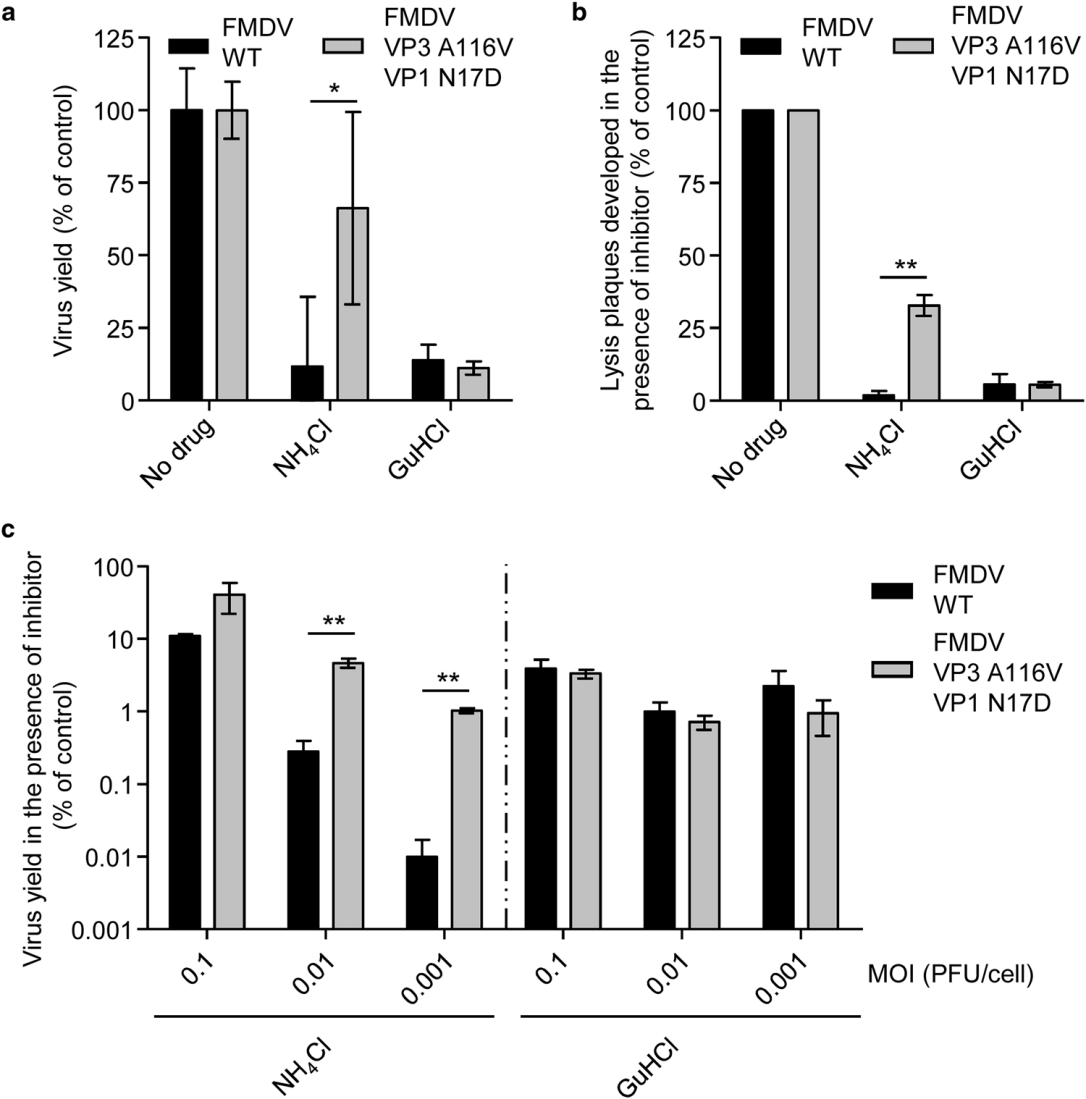
Viral populations carrying VP3 A116V and VP1 N17D replacements display increased resistance to NH_4_Cl but not to GuHCl. (**a**) Analysis of the resistance of FMDV WT and FMDV VP3 A116V + VP1 N17D to NH_4_Cl and GuHCl. BHK-21 cells treated or not with 25 mM NH_4_Cl or 4 mM GuHCl, were infected with FMDV WT or FMDV VP3 A116V VP1 N17D at a MOI of 0.5 PFU/cell. Virus yield at 8 h post-infection was determined by standard plaque assay. Two tailed Student’s *t*-test *P* values between control and drug-treated samples were corrected for multiple comparisons using the Sidak–Bonferroni method (*P < 0.05). Data represent the means ± SDs (*n* = 3–6). (**b**) Plaque assay of FMDV WT and FMDV VP3 A116V + VP1 N17D in the absence or presence of inhibitors. Viral progeny obtained from transfection of viral RNA from infectious clones was titrated in in semi-solidum medium in the absence of drug or the presence of 25 mM NH_4_Cl, 4 mM GuHCl. The number of lysis plaques produced in each condition was determined by crystal violet staining (30 h post-infection) and is expressed as the percentage of PFU developed in presence of the drug relative to that obtained in absence of the drug (*n* = 3–6). (**c**) Effect of MOI on the resistance against NH_4_Cl and GuHCl. Monolayers of BHK-21 cells treated or not with 25 mM NH_4_Cl, 4 mM GuHCl or no inhibitor (control) were infected with FMDV WT or FMDV VP3 A116V + VP1 N17D, at a MOI of 0.1, 0.01 or 0.001 PFU/cell. Virus yield produced in the presence of each drug (8 h post-infection) was determined by standard plaque assay and is expressed as a percentage of that obtained in the absence of inhibitors (control) (n = 3). Two tailed Student’s t-test P values between control and drug-treated samples were corrected for multiple comparisons using the Sidak–Bonferroni method (**P < 0.005). Data represent the means ± SDs.

To test if the increased resistance of FMDV VP3 A116V + VP1 N17D to NH_4_Cl applied also to other FMDV inhibitors that block infection by other mechanisms, we tested the effect of guanidine hydrochloride (GuHCl), a compound that targets RNA dependent RNA polymerase and inhibits FMDV replication^38,39^. While FMDV VP3 A116V + VP1 N17D was less inhibited by NH_4_Cl than FMDV WT, GuHCl inhibited the infection of both viruses to a similar extent (Fig. 4a). These results indicated that resistance of FMDV VP3 A116V + VP1 N17D to NH_4_Cl does not entail a general antiviral resistance.

Next, the frequency of NH_4_Cl and GuHCl resistant variants in FMDV VP3 A116V + VP1 N17D and FMDV WT populations was explored. To this end, the capacity to infect and produce lysis plaques in drug-treated monolayers was analyzed. In these experiments, viral populations directly recovered from transfection of in vitro synthesized viral RNA from the respective infectious cDNA clones, without further amplification, were used, hence minimizing the effects of viral amplification in quasiespecies variability. In the WT population, the percentage of NH_4_Cl resistant variants obtained (1.8%) was in agreement with previous data^11^ (Fig. 4b). However, this percentage was significantly increased (32.7%) in the case of FMDV VP3 A116 + VP1 N17D. In contrast, no significant differences between the resistant lysis plaques of the mutant and the WT were found for GuHCl (about 5% in both cases; Fig. 4b). The results suggest the rapid generation of NH_4_Cl resistant-variants in FMDV VP3 A116V + VP1 N17D populations, a feature not observed for GuHCl resistant-variants. When drug-resistant variants are present at low frequency in the viral population, a lower MOI should negatively affect the expression of the phenotype^40^. The impact of reducing MOI on the degree of resistance against NH_4_Cl and GuHCl was analyzed using viral populations directly recovered from transfection of in vitro synthesized viral RNA (Fig. 4c). The results show that the ability to grow in the presence of NH_4_Cl was negatively affected by lowering MOI. Nevertheless, FMDV VP3 A116V + VP1 N17D was significantly less affected than WT population at all MOI tested, with the difference between these viruses being accentuated at low MOI (Fig. 4c). No effect of decreasing MOI was observed in the case of the treatment with GuHCl. These results suggest that whereas NH_4_Cl-resistant minority variants are present in both FMDV WT and FMDV VP3 A116V VP1 N17D populations they are more represented in the double mutant populations.

### Presence of NH_4_Cl-resistant substitutions in FMDV VP3 A116V + VP1 N17D populations

We have previously described that the pentameric interface of VP3 together with the N terminus of VP1 constitute important hot spots for the selection of mutations that modulate acid sensitivity of FMDV capsid^11,13^. Therefore, the proportion of genomes carrying substitutions previously related with NH_4_Cl resistance in this genomic region was analyzed by NGS (Table 1). For this purpose, FMDV WT and double mutant VP3 A116V + VP1 N17D were subjected or not to a single round of NH_4_Cl treatment. Confirming the validity of the approach, the proportion of genomes encoding VP3 A116V substitution was close to 1 in the double mutant and markedly lower in WT. These analyses indicated that two nucleotide changes (C2985T and A3241G) introducing amino acid replacements VP3 D115E and VP1 T12A previously associated with increased resistance with NH_4_Cl were more represented in double mutant populations treated with NH_4_Cl when compared to WT FMDV grown in the presence or in the absence of NH_4_Cl. In the case of VP3 D115E, this substitution was also more represented in the double mutant populations not subjected to NH_4_Cl treatment (Table 1). Taken together, these results evidence/support the predisposition of mutant VP3 A116V + VP1 N17D to acquire additional mutations leading to increased NH_4_Cl resistance.

**Table 1:** Analysis of mutation frequency by NGS.

Proportion of genomes carrying substitutions previously associated with resistance to NH_4_Cl^a^	FMDV			
	WT		VP3 A116V + VP1 N17D	
	No drug	NH_4_Cl	No drug	NH_4_Cl
C2895A (VP3 D115E)	0	2.1 × 10–4	2.2 × 10–4	4.6 × 10^–4^
G2896T (VP3 A116T)	0	0	0	0
C2897T (VP3 A116V)	2.55 × 10^–3^	3.96 × 10^–3^	0.99	0.99
C2903T (VP3 A118V)	0	0	0	0
G2917A (VP3 A123T)	0	0	0	0
G3238A (VP1 V11I)	0	0	_0_	0
A3241G (VP1 T12A)	0	0	0	5.2 × 10^–4^
C3242T (VP1 T12I)	0	0	0	0
T3259C (VP1 Y18H)	0	0	0	0

^a^The average proportions of each substitution conferring NH4Cl resistance^11,13^ were determined by NGS in three independent experiments (MOI of 0.01 PFU/cell).

### Fitness gain of FMDV VP3 A116V + VP1 N17D is associated with VP1 substitution T22N

**Figure 5:**
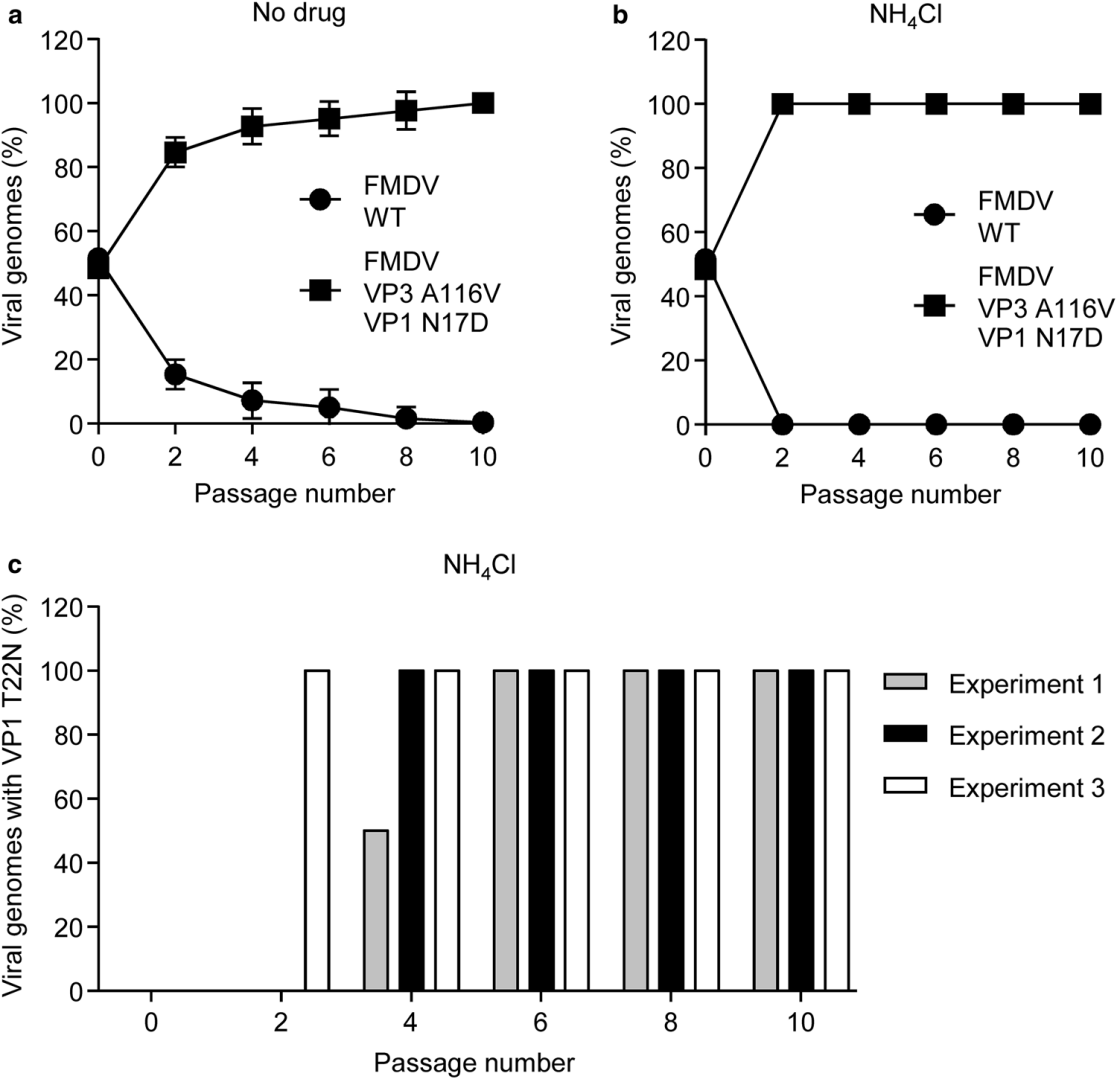
Fitness gain of VP3 A116V + VP1 N17D mutant. (**a**,** b**) Increase in fitness conferred by VP3 A116V + VP1 N17D replacements both in presence and in absence of NH_4_Cl. Competition experiments between WT and double mutant VP3 A116V + VP1 N17D during serial passage of a virus mixture, in the absence (**a**) or in the presence of 25 mM NH_4_Cl (**b**). An initial MOI of 0.1 PFU/cell (0.05 PFU/cell for each virus) was used for each infection. Approximate percentage of competing genomes during serial passages was determined by sequencing the capsid coding cDNA. Passage 0 denote the initial mixture of the viruses. Data represent the means ± SDs (*n* = 3). (**c**) Rapid fixation of VP1 T22N replacement (nucleotide substitution C3272A) in VP3 A116V + VP1 N17D mutant passaged in presence of 25 mM NH_4_Cl. The percentage of genomes carrying nucleotide substitution C3272A was calculated in three independent passage series (Experiments 1, 2 and 3).

Viral fitness measures the capacity to produce infectious progeny^41,42^. To compare the fitness of FMDV WT and FMDV VP3 A116V + VP1 N17D, viral populations directly recovered from infectious clones were tested in competition experiments performed in the absence or presence of NH_4_Cl (Fig. 5a, b). Equal PFU of each of virus populations were mixed, and used to infect cells treated or not with NH_4_Cl. Virus recovered from the initial infection was harvested and further passaged a total of ten times either in absence or presence of NH_4_Cl. The percentage of competing genomes during the passages was determined (Figs. 4b, 5a). In each of the three independent experiments performed in the absence of NH_4_Cl, the genome of FMDV VP3 A116V + VP1 N17D became dominant in the second passage (about 80% of the total viral population) and its proportion progressively increased during passages up to values close to 100% (Fig. 4a). Even more pronounced was the dominance of FMDV A116V + VP1 N17D in each of three independent passage series performed in the presence of NH_4_Cl (about 100% of the total viral population by the second passage) and such dominance was maintained until the tenth passage (Fig. 5b). Sequencing of the capsid-coding region of the populations competing in the presence of NH_4_Cl showed an additional nucleotide change, C3272A, responsible for amino acid substitution T22N in VP1 that rapidly became dominant (between passage 2 and 4) in the three independent passage series (Fig. 5c). No additional mutations were found in the populations competing in the absence of NH_4_Cl. Replacement VP1 T22N has been previously associated with an increase in acid lability of the FMDV capsid^13^, which further supports that the mechanism of adaptation to alkalinized endosomes in NH_4_Cl-treated cells was based on an elevation of the pH threshold for uncoating. These results suggest that the double amino acid replacement A116V in VP3 and N17D in VP1 may facilitate selection of T22N in VP1, a substitution that elevates the pH threshold for uncoating.

Overall, these results show an increase in viral fitness in FMDV VP3 A116V + VP1 N17D that would enable the rapid selection of NH_4_Cl-resistant variants, which relate to the NH_4_Cl-resistance-prone phenotype of FMDV VP3 A116V + VP1 N17D.

## Discussion

The present study has addressed the relationship between acid stability of FMDV capsid and the requirement for endosomal pH acidification for uncoating. We evaluated the resistance to NH_4_Cl of a panel of FMDV variants displaying different acid stability. NH_4_Cl was selected for these studies because our previous work with V-ATPase inhibitors such as concanamycin A has shown that they are less potent FMDV inhibitors than NH_4_Cl maybe because they block the acidification of endosomes while NH_4_Cl neutralizes more efficiently the endosomal pH by acting as a proton sink inside endosomes^11,12^. Unexpectedly, a genetically engineered FMDV that combined amino acid replacements A116V in VP3 and N17D in VP1 exhibited similar acid resistance than the parental FMDV^13^ but displayed increased resistance to NH_4_Cl. This mutant virus combined amino acid replacement VP3 A116V, which confers increased acid lability and hence induces resistance to NH_4_Cl^11,13^, with amino acid replacement VP1 N17D, which provides increased acid stability and hence increases sensitivity to NH_4_Cl^12,17,19^. Interestingly, whereas the combination of both substitutions in a single genome resulted in an additive effect and led to a virus with similar acid sensitivity than WT, this variant showed higher resistance to NH_4_Cl.

The escape from NH_4_Cl is concomitant with a rapid increase in the acid lability of the FMDV VP3 A116V + VP1 N17D population. This change in acid lability could not initially be explained by the selection of a single or a reduced number of mutations in the consensus sequence, suggesting that the mutant cloud composition may be responsible for this phenotypic change. Such effects on fitness recovery have been previously observed for other viral models^43^. Our experiments to analyze the frequency of NH_4_Cl-resistant variants and the effect of decreasing MOI on resistance to NH_4_Cl also supported that although both FMDV WT and FMDV VP3 A116V + VP1 N17D populations showed NH_4_Cl-resistant minority variants, they were 18-fold more represented in the FMDV VP3 A116V + VP1 N17D population. Therefore, it seemed that the presence of the two substitutions VP3 A116V and VP1 N17D might facilitate selection of NH_4_Cl-resistant variants. NGS analyses confirmed/supported this hypothesis for two different nucleotide replacements leading to amino acid substitutions (VP3 D115E and VP1 T12A) previously related to NH_4_Cl resistance. Further passaging in the presence of the NH_4_Cl during competition experiments led to the fixation of an additional amino acid replacement (VP1 T22N) located close to D17 in the N-terminal region of VP1^44^ previously linked to increased acid lability of FMDV capsid, and resistance to NH_4_Cl^13^. These observations suggest that during the treatment with NH_4_Cl, variants such as those with VP3 D115E, VP1 T12A, VP1 T22N may be present at frequencies insufficient to modify the consensus sequence but that can be readily selected by NH_4_Cl.

Our previous work has shown a variety of amino acid substitutions in the FMDV capsid as responsible for NH_4_Cl resistance^11,13^. However, in the three competition experiments between FMDV WT and FMDV VP3 A116V + VP1 N17D, the same amino acid replacement (VP1 T22N) was independently selected. The selection of this amino acid replacement was also previously documented during serial passage of this double mutant in NH_4_Cl in an independent series of experiments^13^. Thus, the tendency for the selection of replacement VP1 T22N points to deterministic constrains for capsid evolution in the presence of NH_4_Cl, probably explained by a selective advantage of VP1 T22N in the context of the VP1 N17D and VP3 A116V double substitution. Regarding the increase in acid resistance as a result of the introduction of VP1 T22N, it was previously proposed that the complexity of the interactions established by the residues located at the N terminus of VP1 with multiple residues located in VP4 and also in VP2 and VP3 could modulate acid-resistance^13^. However, further work should be performed to elucidate the molecular mechanism behind the increase in acid sensitivity induced by this replacement. The results of the competition experiments indicated also that the double mutant exhibited an increase in biological fitness in both the presence and the absence of NH_4_Cl, although it was more obvious in the presence of NH_4_Cl. The lack of resistance of the double mutant to GuHCl, an inhibitor of replication that targets the viral polymerase, indicates that fitness per se, as a multidrug resistance trait documented for hepatitis C virus^40^, is not the mechanism of NH_4_Cl resistance in FMDV VP3 A116V + VP1 N17D. High fitness may facilitate explorations of sequence space to select for substitution VP1 T22N that expresses the resistance phenotype in the context of the double substitution. There is previous evidence that VP1 N17D replacement in its own did not alter biological fitness of FMDV C-S8c1 at neutral pH^12^, nor was it associated with a loss of fitness at neutral pH in another FMDV serotype^19^. However, mutants carrying VP1 N17D together with an additional substitution (H145Y) displayed an increase in viral fitness at neutral pH^17^. This result is in agreement with other evolutionary studies showing that although pathogens that develop resistance to drugs usually have reduced fitness, compensatory mutations that restore fitness may facilitate the stability of the resistance phenotype^45,46^.

Viral capsids are metastable assemblies evolved to protect the genome while allowing its release for infection^1^. Upon acidification, the FMDV capsid undergoes a profound and irreversible conformational alteration that leads to its dissociation into pentameric subunits. We have previously demonstrated that other amino acid substitutions similar to VP3 A116V, such as VP3 A118V, facilitate capsid dissociation through the introduction of bulkier amino acid side chains close to the pentameric interfaces, thus debilitating interpentameric interactions^11^. However, this effect was counteracted by amino acid substitutions that stabilize the capsid^13^. In this way, a balance between stability and dissociability should be conserved by both WT and double mutant FMDV VP3 A116V + VP1 N17D. Our data suggest that this double mutant could displace this balance towards the generation of acid-labile populations more easily than FMDV WT. Therefore, the fitness increase of the double mutant together with a lower threshold for destabilization could explain the increased ability to produce acid-labile capsid of the double mutant population and hence to escape from the inhibitory effect of NH_4_Cl. From an evolutionary perspective, the results show how the presence of specific combination of mutations in a viral genome can modify phenotypes in unpredictable manners and the capacity of a virus to respond to a selective constraint.

In summary, the present study provides novel evidences showing that the combination of mutations with opposite effects on acid-stability can result in compensatory effects that can lead to an unforeseen fitness gain, and facilitate rapid adaptation against an inhibitor of acid-dependent uncoating.
